# Development of a questionnaire to measure primary care physicians’ scope of practice

**DOI:** 10.1186/s12875-015-0357-z

**Published:** 2015-11-02

**Authors:** Kenya Ie, Shuhei Ichikawa, Yousuke C. Takemura

**Affiliations:** Department of Family Medicine, Mie University School of Medicine, 2-174 Edobashi, Tsu, Mie 514-8507 Japan; Graduate School of Public Health, University of Pittsburgh, 130 De Soto Street, Pittsburgh, PA 15261 USA

**Keywords:** Comprehensive health care, Primary health care, Family practice, Physician’s practice patterns, Questionnaires, Validation studies

## Abstract

**Background:**

Despite an increase in research devoted to primary care attributes, the patient benefits and educational aspects of broad scope practice of primary care physicians (PCPs) have not been well studied, due to a lack of validated measurement in each country. The objective of this study was to develop and validate the Scope of Practice Inventory (SPI) to measure physicians’ scope of practice within the Japanese primary care setting.

**Methods:**

The questionnaire was developed in seven phases: 1) item generation, 2) consensus method for necessity of each item, 3) Delphi process for the importance of each item, 4) pilot tests to limit the number of items, 5) preliminary cross-sectional study to examine factor structure and to validate the construct validity, 6) evaluation of internal consistency and intra-class reliability, and 7) evaluation of external validity. To confirm the interpretability of the SPI, the determinants of the SPI using a generalized linear model were evaluated.

**Results:**

Among 359 items generated by a focus group, 180 reached a defined consensus on face and content validity after the Delphi process. After deletion of items with Kappa values less than 0.6, 120 items were selected for the preliminary study. The principle component analysis using responses from 451 PCPs eliminated 52 items. The final 68-point SPI had three subdomains: Inpatient care, 25 items; Urgent care and minor procedures, 27 items; and Ambulatory care, 16 items. Internal consistency and test-retest reliability for total SPI and each subdomain revealed acceptable reliability. Male sex, less years since graduation, working in a hospital, sub-urban or rural setting, having remote experience, and having board certification as a PCP were positively associated with higher SPI.

**Conclusions:**

We developed a self-administered 68-point scale, the SPI, which had satisfactory validity and reliability. Primary care quality and educational research using SPI are expected to contribute to comprehensive and efficient health care systems in the future.

**Electronic supplementary material:**

The online version of this article (doi:10.1186/s12875-015-0357-z) contains supplementary material, which is available to authorized users.

## Background

According to OECD Health Statistics 2013, Japan has achieved the second highest life expectancy in the world, with an average ratio of national health expenditure against GDP [1]. However, Japanese society has been predicted to face an extraordinary rapid aging rate in the next several decades, to the extent that approximately 40 % of people will be 65 years or older in 2060 [2]. Thus, achieving effective primary care for this multimorbidity population, while capping on medical expenses increase, is of great interest.

Several studies have reported a positive relationship between primary care physician (PCP) supply and better health outcomes [3, 4]. For practical purposes, perception of primary care attributes accounts for appropriate use of resources, and patient outcomes are indispensable for quality care. Based on the 1978 Alma-Ata Declaration, the World Health Organization has proposed a global goal of achieving universal primary care with the following attributes: first contact care, person-centredness, comprehensiveness and integration, continuity of care, responsibility, and coordination [5]. Among these attributes, the influence of person-centredness [6], continuity [7, 8], and coordination [9] to health care outcomes have been well demonstrated. On the other hand, there are few studies about the benefits of comprehensiveness, although the fact that family physicians cover a broader scope of practice compared to specialists has been well accepted.

A major challenge has been to break down the complicated concept of comprehensiveness into components that can be measured. According to the previous articles, the definition of ‘comprehensiveness’ roughly consisted of two groups [10]. The first group corresponds comprehensiveness to scope*; “a spectrum of care that covers a broad range of patient needs*” [11]. Starfield had proposed the following three dimensions on which to measure PCPs’ comprehensiveness: 1) range of diagnoses made; 2) range of services covered; and 3) percent of patients referred [4]. Among these dimensions, the range of services covered by PCPs has been the most commonly used. The second group referred comprehensiveness to a broader meaning including the whole-person care aspect [5, 10]. Whole-person care refers to ‘relational-continuity’ , ‘interpersonal communication’ and ‘patient-centered care’ , for which it was far more difficult to set an appropriate definition that can be measured. With respect to our research, we decided to clearly focus on family physicians’ scope of practice (e.g., diagnoses made, services covered, and procedures performed).

Although ‘comprehensiveness/scope of practice’ measurements have existed as a questionnaire [12] or as subdomains of questionnaires [13–15] in several countries, differences in the health care system and the delivery of primary care services make a simple application of foreign scales difficult. Furthermore, these existing tools were designed to feasibly evaluate comprehensiveness as one aspect among several attributes of PCP. The limited number of items about the scope of practice among these scales has resulted in limited accuracy as an indicator, and has made its application difficult for broader utilization, such as evaluation of PCP residency/medical education achievement, continuous medical education index for PCPs, and the evaluation of balance of supply and demand regarding specific health services for each region.

The purpose of this study was to establish and to validate a self-reported questionnaire (the Scope of Practice Inventory (SPI)) to specifically measure physicians’ scope of practice, which is applicable and feasible for use in the Japanese primary care setting.

## Methods

### Study design

The questionnaire was developed in seven phases: 1) A literature review, followed by a focus group with seven PCPs for the purpose of item generation, 2) a consensus method for necessity of each item, 3) a Delphi process with six other PCPs to gain consensus regarding the importance of each item and to evaluate the content validity of the questionnaire, 4) pilot tests to limit the number of items, 5) a preliminary questionnaire-based cross-sectional study to validate the construct validity of the questionnaire, 6) evaluation of internal consistency and intra-class reliability, and 7) evaluation of external validity.

All processes of the questionnaire development and validation were compliant with the COSMIN checklist [16]. Written informed consent was obtained from all participants except in Phase 5. In Phase 5, anonymous responses were collected from participants who read the informed consent statement and voluntarily responded in the survey. The study protocol was approved by the Institutional Ethical Committee of Mie University Graduate School of Medicine (No. 1219).

### Phase 1: focus group for item generation

The research team conducted a literature review to identify the basis for the question items using several evaluation criteria (applicability in Japanese primary care setting, comprehensiveness, measurability, based on consensus decision-making in multiple stakeholders, and published after 2000) and retrieved three potential item pools: International Classification of Primary Care, Second Edition [17], Recommended Curriculum Guidelines for Family Medicine Residents by the American Academy of Family Physicians [18], and Curriculum for Continuous Medical Education (Japan Medical Association 2009) [19]. Based on these instruments, the research team gathered items after eliminating overlaps.

A focus group with seven PCPs was then undertaken. Physicians with a good deal of knowledge regarding PCPs’ competencies were recruited specifically for their populations served (urban, sub-urban, and rural), practice settings (clinic, small-scale hospital, and large-scale hospital), and years since graduation (6–10 years, 11–15 years, and more than 15 years). These experts included two PCPs from private clinics in both urban and rural settings, two PCPs from rural small-scale hospitals, two general internists from tertiary hospitals in both urban and sub-urban settings, and an academic clinician from an urban university hospital. They included three PCPs with 6–10 years’ , three with 11–15 years’ , and one with more than 15 years’ experience (mean = 13.1, SD = 6.8). The experts were asked to review the item list and to add items that they believed were necessary. They then rewrote items if they were ambiguous.

### Phase 2: consensus method for necessity of each item

On November 2011, seven PCPs who enrolled in Phase 1 rated each item generated through the focus group using a three-point Likert scale (1, necessary; 2, do not know; 3, unnecessary) regarding the item’s necessity as a competency of PCPs. Consensus was defined as six out of seven participants having the same opinion regarding an item. The distribution of the participants’ responses was revealed during each voting round, and revisions were made following a group discussion focused on the necessity and face validity of each item. The participants re-ranked the items for which there had not been consensus or which were rewritten for three rounds in total.

### Phase 3: Delphi process for importance of items

From February to March 2012, a four-step Delphi process was used for item selection. The expert panel of six PCPs included two general internists working in large-scale hospitals, two small-scale hospital-based PCPs, and two clinic-based PCPs; in each of these three pairs of physicians, one physician came from a rural or sub-urban practice and the other from an urban practice. The PCPs years since graduation were ranged between 6 and 29 years (mean = 13.2, SD = 8.5). Group discussion and ranking was focused on “Do you agree that this item is important for measuring PCPs’ scope of practice?” The items were assessed using a nine-point Likert scale regarding the importance of each item ranging from 1 (strongly disagree) to 9 (strongly agree). The feedback given on each item in previous rounds was reported during each round of voting, and revisions were made based on a group discussion focused on the face validity of each item. Consensus was defined as all participants having ranked an item within a range of three consecutive numbers.

### Phase 4: construction of preliminary questionnaire

From June 2012 to July 2012, 33 PCPs, each of whom remained in the same facility in Mie prefecture (whether clinic, small-scale hospital, or large-scale hospital) for the preceding year were recruited for a preliminary web-form survey to ensure the feasibility and reliability of the questionnaire. Those who regularly practice at more than one facility were asked to respond for each setting separately. The list of dual-forced items obtained through the Delphi process, which included “do” or “don’t do” the item at one’s current practice was used. The participants were asked to repeat the questionnaire two times at a thirty minute interval in order to identify obscure or confusing items; items with kappa scores less than 0.6 were excluded to limit the number of items and to ensure reliability of the preliminary questionnaire.

### Phase 5: questionnaire-based cross-sectional study

A cross-sectional web-form-based study was conducted using a preliminary questionnaire in February 2013. Nine hundred and sixty-nine Japanese PCPs with the same distribution of attributes (sex, practice setting, and main practice prefecture) as typical Japanese PCPs were purposefully recruited using an internet research panel list. To investigate the construct validity of the questionnaire, principal component analysis for categorical variables [20, 21] was conducted. The number of principle components was set by using a scree plot. Varimax rotation was used to obtain the final version [22]. Items with factor loading below 0.5 or above 0.5 for two or more domains were eliminated from the preliminary version of the SPI. The process of validation was confirmed after agreement on the interpretability of the factors was reached among the research team. In the final version of the SPI, each item was ranked in order of its factor loading value.

### Phase 6: evaluation of reliability

Internal consistency was analyzed by calculating Cronbachs’ alpha for total score and each subdomain among the cross-sectional survey sample. The cutoff for a Cronbachs’ alpha was set at 0.70.

A test-retest survey following the principle component analysis was conducted to test intraclass reliability. From January 2015 to February 2015, we sent a questionnaire mail survey request to 42 PCPs. The respondents were restricted to answering only items that addressed topics they had ‘actually experienced’ at their current facilities ever before. The participants were asked to answer sets of the same questionnaires two weeks apart, and estimates of the intraclass correlation coefficient between two sets of scores for overall SPI and each subdomain were assessed. The cutoff for an intraclass correlation coefficient was set at 0.70, which was considered to be acceptable for test-retest reliability.

### Phase 7: evaluation of external validity

Since there was no clearly set gold standard for a Japanese PCP’s scope of practice, criterion-related validity was analyzed by correlating the visual analogue scale of the physician’s subjective ‘comprehensiveness’ with the overall SPI score. Inter-factor correlation was calculated to evaluate the association of each subdomain. To confirm the interpretability and the characteristic of the final version of the SPI, we calculated means and 95 % confidential intervals of SPIs for each of the following stratifications: sex, age, post-graduate years, practice setting, population served, having any working experience in the remote setting, and having board certification as a family physician or a primary care physician in Japan. Additional analyses were conducted to preliminarily show the determinants of the scope of practice, by using a generalized linear model (GLM). Because the SPI is determined by the number of “Experience” responses, we assumed the distribution of the SPI as poisson, and used log as link-function. “Post-graduate years” was treated as a continuous variable, although it is shown in a stratified manner in Table 5. The other independent variables were dummied, and their criteria are shown in Table 5. “Age” was omitted from the GLM analysis to avoid multicolinearity.

## Results

### Construction of preliminary questionnaire (Phase 1 ~ 4)

Among 359 items (e.g., symptoms, diseases, procedures) generated by the focus group, a consensus regarding “necessity” was reached on 216 items through the consensus method. Subsequent Delphi process extracted 180 preliminary item pools related to scope of practice with good face validity that were thought to be important for PCPs. A total of 180 dual-forced items were included on the web-form survey, and a cumulative total of 40 responses was received from 29 PCPs (a response rate of 88 %). This included 15 clinic-based PCPs, 15 small-scale hospital PCPS, and 10 large-scale hospital PCPs. Among them, 11 PCPs worked in urban, 23 in sub-urban, and 6 in rural areas. The mean years since graduation was 13.4 (SD = 6.66, range 5–29). After excluding 60 items with Kappa values lower than 0.6, 120 dichotomous items were selected for the preliminary questionnaire.

### Principal component analysis (Phase 5)

#### Participants demographics

**Table 1:** Sociodemographic characteristics of sampled physicians in Phase 5 survey (*N* = 451)

Characteristics	Respondents	*N*	%
Sex	Male	378	83.80 %
	Female	73	16.20 %
Age (years)	<35	30	6.70 %
	35–44	89	19.70 %
	45–54	196	43.50 %
	55–64	119	26.40 %
	≧65	17	3.80 %
Years since graduation	0 ~ 10	34	7.50 %
	11 ~ 20	112	24.80 %
	21 ~ 30	219	48.60 %
	31 ~ 40	77	17.10 %
	41~	9	2.00 %
Practice setting	Clinic	258	57.20 %
	Hospital (<100 beds)	60	13.30 %
	Hospital (≧100 beds)	133	29.50 %
Population served	Urban	280	62.10 %
	Sub-urban	144	31.90 %
	Rural	27	6.00 %
Experience in remote settings	Yes	135	29.90 %
	No	316	70.10 %

Among 969 physicians who received the preliminary questionnaire, 451 (46.5 %) responded. Distribution of the participants’ gender, age group, years since graduation, practice setting (hospital or clinic), poplation served (urban, sub-urban, or rural), and experience in remote rural settings are shown in Table 1. Due to stratified sampling, these distribution were thought to resemble that of typical Japanese PCPs.

#### Psychometric analysis

Based on the scree plot, the number of principle components was set at three. Among 120 items on the preliminary questionnaire, 52 items with factor loading below 0.5 or above 0.5 for two or more domains were eliminated. Table 2 shows the factor loadings of the SPI after varimax rotation. The three subdomains were named as “Inpatient care”, “Urgent care and minor procedures”, and “Ambulatory care”, and 25, 27, and 16 items were included in each domain, respectively.

**Table 2:** Final rotated factor loadings for SPI items

Items	Factor 1	Factor 2	Factor 3
	*Inpatient care*	*Urgent care and minor procedures*	*Ambulatory care*
Inserting a nasogastric tube	**0.80**	0.13	0.01
Performing blood transfusion	**0.77**	-0.02	-0.04
Deciding to apply gastrostomy to patients with recurrent aspiration	**0.77**	0.08	0.15
Performing thoracocentesis	**0.75**	0.05	-0.07
Performing paracentesis	**0.74**	0.11	0.01
Collecting and evaluating atrial blood gas	**0.74**	-0.06	-0.01
Intra-tracheal intubation	**0.71**	0.17	0.03
Managing parenteral nutrition	**0.68**	0.14	0.13
Exchanging enteral feeding tube and managing feeding tube problems	**0.68**	0.15	0.09
Ventilating a patient with respiratory failure using bag valve mask	**0.68**	0.22	0.05
Use of opioids for terminal patients	**0.67**	0.22	0.15
Caring for symptoms other than pain for terminal patients	**0.65**	0.21	0.18
Pain management for terminal patients using VAS score	**0.64**	0.25	0.11
Interpreting brain CT scan	**0.64**	-0.05	0.10
Terminal care for non-malignant patients	**0.62**	0.23	0.26
Inserting urinary tract catheter	**0.61**	0.26	0.09
Initial treatment for shock state patients	**0.60**	0.23	0.26
Explaining a terminal stage patient's condition to family	**0.59**	0.20	0.32
Performing intravenous sedation and pain management	**0.59**	0.27	0.05
Initial diagnostic approach for patients with disturbance of consciousness	**0.59**	0.21	0.22
Providing counseling about life-prolonging treatment	**0.58**	0.22	0.24
Diagnosing and treating delirium	**0.57**	0.27	0.21
Evaluating the necessity and performance of lumbar puncture	**0.56**	0.27	-0.05
Interpreting brain MRI	**0.55**	0.01	0.14
Initial diagnosis and management for stroke	**0.54**	0.19	0.33
Splinting for sprain	0.24	**0.71**	0.03
Manipulative reduction of radial head subluxation	0.15	**0.70**	0.03
Diagnosing and managing burns	0.13	**0.69**	0.08
Advising on daily care for musculoskeletal problems	0.11	**0.68**	0.14
Diagnosing and managing osteoarthritis of the knee	0.14	**0.67**	0.22
General advice for parents of children with fever	-0.06	**0.66**	0.24
Initial care for animal/human bite and follow-up	0.25	**0.66**	0.16
Performing knee arthrocentesis	0.16	**0.66**	0.03
Initial treatment of simple fracture (splinting)	0.37	**0.65**	-0.06
Diagnosing and treating acute monoarthritis	0.13	**0.63**	0.21
Performing trigger point injection	0.10	**0.63**	0.07
Examining external auditory canal and tympanic membrane using otoscope	0.09	**0.62**	0.06
Peripheral venous access for pediatric patients	0.07	**0.62**	0.06
Ordering intravenous fluid for pediatric patients	0.01	**0.62**	0.11
Deciding to apply bust band for chest trauma	0.26	**0.61**	0.05
Deciding if a chest x-ray is indicated in pediatric patients	-0.05	**0.60**	0.15
Hemostasis for superficial bleeding	0.24	**0.60**	0.07
Diagnosing and treating scapula-humeral periarthritis	0.01	**0.59**	0.28
Examining anterior eye without equipment	0.18	**0.58**	0.10
Hemostasis for nasal bleeding	0.29	**0.58**	0.11
Diagnosing and treating acute otitis media	0.15	**0.57**	0.18
Performing digital block	0.23	**0.56**	-0.05
Suturing cut wounds	0.36	**0.56**	-0.06
Initial management of febrile seizure	0.08	**0.55**	0.07
Removing earwax or foreign body from external ear canal	0.14	**0.54**	0.03
Diagnosing skin eruption	0.20	**0.53**	0.25
Advising for skin care	0.05	**0.51**	0.23
Diagnosing and managing bronchial asthma	0.05	0.15	**0.64**
Diagnosing and managing diabetes	0.10	-0.03	**0.63**
Diagnosing and managing dyslipidemia	0.04	-0.04	**0.60**
Diagnosing and managing hypertension	0.02	-0.07	**0.60**
Diagnosing and managing hyperuricemia	-0.05	-0.04	**0.59**
Diagnosing and managing thyroid dysfunction	0.10	0.10	**0.59**
Diagnosing and managing insomnia / sleep disturbance	0.07	0.12	**0.58**
Treating urinary tract infection	0.18	0.09	**0.57**
Diagnosing and managing chronic obstructive pulmonary disease	0.29	0.18	**0.56**
Diagnosing and managing allergic rhinitis	-0.08	0.19	**0.55**
Diagnosing and managing urticaria / angioedema	0.13	0.27	**0.55**
Diagnosing and determining the urgency of headache	0.19	0.19	**0.54**
Appropriate management of hematuria	0.09	0.24	**0.54**
Diagnosing and determining the urgency of dizziness	0.14	0.20	**0.54**
Outpatient management of heart failure	0.27	0.18	**0.53**
Diet therapy in the outpatient encounter	0.21	0.18	**0.53**

The bold data represent factor loadings greater than 0.5

### Evaluation of reliability (Phase 6)

Table 3 reveals the descriptive statistics, Cronbachs’ alpha coeffcients, and intraclass correlation coefficients for total SPI and each subdomain. Cronbachs’ alpha values for total SPI, ‘Inpatient care’ , ‘Urgent care and minor procedures’ , and ‘Ambulatory care’ were 0.96, 0.95, 0.94, and 0.87, respectively, which all satisfied acceptable internal consistency.

**Table 3:** Descriptive statistics and reliability of the SPI and each subdomain (*n* = 451)

SPI/Subdomains	Mean (SD)	Range	Cronbachs’ alpha	Intraclass correlation coefficient
SPI (total)	36.00 (15.67)	0–68	0.96	0.96
Inpatient care	11.93 (8.31)	0–25	0.95	0.97
Urgent care^a^	9.85 (8.09)	0–27	0.94	0.96
Ambulatory care	14.22 (2.88)	0–16	0.87	0.87

^a^Urgent care and minor procedures

With regard to test-retest reliability, 34 out of 42 physicians (81.0 %) who were requested to enroll in the survey responded. The intraclass correlation coefficients for total SPI, ‘Inpatient care’ , ‘Urgent care and minor procedures’ , and ‘Ambulatory care’ were 0.96, 0.97, 0.96, and 0.87, respectively. Thus, acceptable reproducibility of the SPI and each subdomain have been demonstrated (Table 3).

### Criterion-related validity (Phase 7)

The criterion-related validity of the overall questionnaire score using the visual analogue scale of the physician’s subjective ‘comprehensiveness’ revealed a significant correlation (Pearson’s correlation coefficient 0.34 (*p* = 0.03)).

### Charasteristics of SPI (Phase 7)

Each subdomain score was moderately correlated with the other subdomains, as shown in Table 4. The demographic data and means, standard deviations, and 95 % confidential intervals of the SPI for each strata are shown in Table 5. Simultaneously, the results of additional GLM analyses are shown in Table 5. All of the factors shown in Table 5 were significant determinants of SPI. Physicians working in a hospital, working in a sub-urban or rural setting, having remote rural experience, and having board certification as a family physician or primary care physician, were positively associated with a higher SPI, whereras being a female phyisician and having longer years since graduation were associated with a lower SPI score.

**Table 4:** Inter-correlation matrix of the questionnaire subdomains (*n* = 451)

Subdomains	Inpatient care	Urgent care^a^	Ambulatory care
Inpatient care	1.00	0.47†	0.50†
Urgent care^a^	0.47†	1.00	0.53†
Ambulatory care	0.50†	0.53†	1.00

†Spearman’s correlation coefficient, all significant at *p* < 0.01

^a^Urgent care and minor procedures

**Table 5:** Demographics and possible determinants of physician’s scope^a^

Items	SPI_mean_	SPI_SD_	estimate	S.E.	z value	*p* value	GVIF
Intercept	NA	NA	3.629	0.044	81.938	<0.001	-
Sex							
Male (criterion)	36.9	16.04					
Female	31.3	12.71	−0.154	0.024	−6.470	<0.001	1.108
Age (years)^b^							
−35	42.0	16.93					
35–44	38.5	16.55					
45–54	34.7	15.06					
55–64	34.8	15.42					
65–	35.7	15.00					
Post-graduate years^c^			−0.003	0.001	−3.446	<0.001	1.207
0–10	43.3	16.60					
11–20	37.3	16.82					
21–30	35.2	15.16					
31–40	33.9	14.31					
41-	32.0	14.65					
Practice setting							
Clinic (criterion)	32.0	14.78					1.184
Hospital (<100 beds)	43.9	14.80	0.283	0.023	12.421	<0.001	
Hospital (> = 100 beds)	40.3	15.34	0.218	0.019	11.658	<0.001	
Population served							
Urban (criterion)	34.3	15.19					1.199
Sub-urban	36.1	15.75	0.060	0.017	3.453	<0.001	
Rural	53.0	8.59	0.301	0.031	9.727	<0.001	
Remote experience							
Yes	43.1	16.66	0.125	0.018	6.747	<0.001	1.275
No (criterion)	33.0	14.22					
Specialist in FM or PC^d^							
Yes	50.8	13.56	0.259	0.028	9.231	<0.001	1.122
No (criterion)	34.9	15.27					

^a^Generalized linear model (GLM) analysis was conducted. Dependent variable was the total score of SPI. We assumed the distribution of the total score of SPI as poisson, and link function was log

Null deviance was 3303.0 (df = 450)/Residual deviance was 2565.4 (df = 442)

AIC was 4973.3

^b^Omitted from GLM analysis, to avoid the occurence of multicolinearity

^c^Treated as numerical variable in GLM analysis

^d^Having board certification as family physician or primary care physician in Japan

## Discussion

We have developed and validated the SPI, a novel physician-administered questionnaire for scope of practice of Japanese PCPs, composed of 68 binary questions. The SPI has several advantages as a questionnaire; for example, the face and criterion-related validity, internal consistency, and test-retest reliability of this physician-administered questionnaire have been well demonstrated. Furthermore, the large number of samples included in the principle component analysis reinforces the power of the analysis. Another advantage is that the SPI is designed to reduce uncertainty related to a questionnaire survey. It has been revealed that patients could only assess their physicians’ scope of practice based on their individualized experience. Thus, obtaining information about the scope of practice from physicians, rather than patients, could be more valid [10]. Therefore, the SPI’s physician-administration format enables more valid evaluation of the scope compared to a patient-administration survey. In addition, the SPI is designed to minimize the social desirability bias related to self-completion. By measuring physicians’ achievements, not their capabilities, with use of a clear definition of “do” as respondents’ actual experience at their own facilities, we have controlled the social desirability bias.

The definition of ‘scope of practice’ within our research was clearly set at the first two of three components among Starfield’s classification of PCPs’comprehensiveness measurements: 1) range of diagnoses made, 2) range of services covered, and 3) percent of patients referred [4]. It is true that an appropriate referral to specialists is a vital function of comprehensive primary care. However, given that referral is also a component of “coordination” among six key attributes of primary care defined by the WHO [5], incorporating referral into the scope of practice would weaken the SPI’s application as an independent questionnaire. A more broad definition, the whole-person care aspect, was also excluded from our definition to avoid ambiguity. Thus, we selectively included symptoms, diagnosis and procedures which PCPs commonly are able to handle.

Trans-cultural utilization of foreign scales was thought to be inadequate because of the differences in health care systems. Several unique characteristics of the Japanese health care system including universal health insurance coverage, free access to health care, and no government accreditation of providing primary care, has lead to non-uniformity among PCPs. Thus, compared to other countries where primary care systems have been well developed, applying foreign PCP scales within Japan is especially difficult. Although there are several articles that have tried to measure the scope of practice among Japanese PCPs [23, 24], no validated questionnaire that can be applied to measuring scope of practice has been published to date. Therefore, a scale suitable for the Japanese primary care setting was needed.

The other reason we developed the SPI instead of translating existing tools from other countries [12–15] was to broaden its usage applications for future research and education related to primary care. In the subdomain of Primary Care Assessment Tools, one of the most widely accepted primary care questionnaires, comprehensive care is measured from services available (11 different types of services, e.g., family planning) and services provided (five age-relevant services, e.g., discussions of ways to stay healthy) from both patient and provider perspectives [13, 15]. Likewise, research from Canada has measured the scope of practice score by the number of medical services provided out of 12 office-based and non-office-based services (e.g., anesthesia, inpatient hospital care) [12]. Despite their advantage as validated, comprehensive and feasible questionnaires, the limited number of scope of practice items has weakened their discrimination capacity for quantification of the scope. The SPI, which is more accurate in terms of measuring the scope of PCPs’ practice, is also applicable to a broad range of practice settings because it consists of three subdomains that enable application of each subscale to different primary care settings. Furthermore, due to its accuracy, good reliability and face validity, the SPI can be applied to residency/medical education achievement assessment, the index of continuous medical education, and to the evaluation of specific health service capability within a medical administration area.

The fact that the SPI contains fewer health maintenance items (e.g., no items on well exam, counseling, or disease prevention), fewer obstetric and gynecological diagnostic procedures, and many inpatient-care-related items compared to previous scales from the western countries [12, 25] would reflect the peculiarity of the Japanese primary care system.(e.g., the universal annual health examination system and the absence of a formal gatekeeping system.) Neverthless, the following variables showed similar effects on scope of physician compared to studies from other countries: rural practice setting, male gender, and younger age [12, 26–28]. These common variables could correspond to multilateral commonality of primary care internationally, although our additional analysis was a preliminary exploratory survey with a relatively small sample size. Further studies will be needed to confirm if such universal features remain internationally.

### Limitations

This study has some limitations. First, the questionnaire still has a relatively large number of items which may render its feasibility, even though the average time to complete the SPI was approximately ten minutes. Second, the assessment of the scope of practice based on the provider’s experience could have a risk of recall bias. There is some possibility of an upward trend of the score that is greater than what would be expected from actual achievements because of the Hawthorne effect. Finally, scope of practice from the providers’ viewpoint is still only an aspect of the complex components of “comprehensiveness”.

## Conclusions

In conclusion, we developed a self-administered 68-point scale, the SPI, which had satisfactory validity and reliability (Additional file 1: SPI English version. Additional file 2: SPI Japanese version). Future studies that apply the SPI in a primary care setting would be expected to clarify factors related to better quality of care, and to be utilized for educational and policy making purposes. These factors could contribute to the countries that will experience the same aging problems as Japan in the near future.

## Additional files

Additional file 1:
**The Scope of Practice Inventory (English version).** (DOCX 148 kb) Additional file 2:
**The Scope of Practice Inventory (Japanese version).** (DOC 110 kb)

## Abbreviations

PCP: Primary care physician
SPI: Scope of practice inventory
GLM: Generalized linear model
