# Supplementary material for: Development of a questionnaire to measure primary care physicians’ scope of practice
Source: BMC Fam Pract. 2015 Nov 2;16:161. doi: 10.1186/s12875-015-0357-z (PMC4630913; doi:10.1186/s12875-015-0357-z)
Supplement: Additional file 1: — The Scope of Practice Inventory (English version). (DOCX 148 kb) [file 12875_2015_357_MOESM1_ESM.docx]

The Scope of Practice Inventory (SPI English version)

This survey investigates your scope of practice in your current practice setting and will take about 5 to 10 minutes to complete. Please read the directions carefully and answer the questions completely and honestly.

Age: Gender: Date:

︎◼Please select items that you “actually do or have done at your current practice” among the following 25 items. Please select the checkbox “Do,” if you actually do or have done the item at your current practice. Please select “Don’t do,” if you have not done the item at your current practice or if you do not understand what the item means.

*Please note to select “Don’t do” if you have not done the item at your “current” practice, even if you think you are competent to do the corresponding item, or have had the experience at past practice settings.

| Items | Do | Don’t do |
| --- | --- | --- |
| A1: Inserting nasogastric tube | □ | □ |
| A2: Performing blood transfusion | □ | □ |
| A3: Deciding to apply gastrostomy to patients with recurrent aspiration | □ | □ |
| A4: Performing thoracocentesis | □ | □ |
| A5: Performing paracentesis | □ | □ |
| A6: Collecting and evaluating atrial blood gas | □ | □ |
| A7: Intra-tracheal intubation | □ | □ |
| A8: Managing parenteral nutrition | □ | □ |
| A9: Exchanging enteral feeding tube and managing feeding tube problems | □ | □ |
| A10: Ventilating a patient with respiratory failure using bag valve mask | □ | □ |
| A11: Use of opioids for terminal patients | □ | □ |
| A12: Caring for symptoms other than pain for terminal patients | □ | □ |
| A13: Pain management for terminal patients using VAS score | □ | □ |
| A14: Interpreting brain CT scan | □ | □ |
| A15: Terminal care for non-malignant patients | □ | □ |
| A16: Inserting urinary tract catheter | □ | □ |
| A17: Initial treatment for shock state patients | □ | □ |
| A18: Explaining a terminal stage patient's condition to family | □ | □ |
| A19: Performing intravenous sedation and pain management | □ | □ |
| A20: Initial diagnostic approach for patients with disturbance of consciousness | □ | □ |
| A21: Providing counseling about life-prolonging treatment | □ | □ |
| A22: Diagnosing and treating delirium | □ | □ |
| A23: Evaluating the necessity and performance of lumbar puncture | □ | □ |
| A24: Interpreting brain MRI | □ | □ |
| A25: Initial diagnosis and management for stroke | □ | □ |

◼Please select items that you “actually do or have done at your current practice” among the following 27 items. Please select the checkbox “Do,” if you actually do or have done the item at your current practice. Please select “Don’t do,” if you have not done the item at your current practice or if you do not understand what the item means.

*Please note to select “Don’t do” if you have not done the item at your “current” practice, even if you think you are competent to do the corresponding item, or have had the experience at past practice settings.

| Items | Do | Don’t do |
| --- | --- | --- |
| B1: Splinting for sprain | □ | □ |
| B2: Manipulative reduction of radial head subluxation | □ | □ |
| B3: Diagnosing and managing burns | □ | □ |
| B4: Advising on daily care for musculoskeletal problems | □ | □ |
| B5: Diagnosing and managing osteoarthritis of the knee | □ | □ |
| B6: General advice for parents of children with fever | □ | □ |
| B7: Initial care for animal/human bite and follow-up | □ | □ |
| B8: Performing knee arthrocentesis | □ | □ |
| B9: Initial treatment of simple fracture (splinting) | □ | □ |
| B10: Diagnosing and treating acute monoarthritis | □ | □ |
| B11: Performing trigger point injection | □ | □ |
| B12: Examining external auditory canal and tympanic membrane using otoscope | □ | □ |
| B13: Peripheral venous access for pediatric patients | □ | □ |
| B14: Ordering intravenous fluid for pediatric patients | □ | □ |
| B15: Deciding to apply bust band for chest trauma | □ | □ |
| B16: Deciding if a chest x-ray is indicated in pediatric patients | □ | □ |
| B17: Hemostasis for superficial bleeding | □ | □ |
| B18: Diagnosing and treating scapula-humeral periarthritis | □ | □ |
| B19: Examining anterior eye without equipment | □ | □ |
| B20: Hemostasis for nasal bleeding | □ | □ |
| B21: Diagnosing and treating acute otitis media | □ | □ |
| B22: Performing digital block | □ | □ |
| B23: Suturing cut wounds | □ | □ |
| B24: Initial management of febrile seizure | □ | □ |
| B25: Removing earwax or foreign body from external ear canal | □ | □ |
| B26: Diagnosing skin eruption | □ | □ |
| B27: Advising for skin care | □ | □ |

◼Please select items that you “actually do or have done at your current practice” among the following 16 items. Please select the checkbox “Do,” if you actually do or have done the item at your current practice. Please select “Don’t do,” if you have not done the item at your current practice or if you do not understand what the item means.

*Please note to select “Don’t do” if you have not done the item at your “current” practice, even if you think you are competent to do the corresponding item, or have had the experience at past practice settings.

| Items | Do | Don’t do |
| --- | --- | --- |
| C1: Diagnosing and managing bronchial asthma | □ | □ |
| C2: Diagnosing and managing diabetes | □ | □ |
| C3: Diagnosing and managing dyslipidemia | □ | □ |
| C4: Diagnosing and managing hypertension | □ | □ |
| C5: Diagnosing and managing hyperuricemia | □ | □ |
| C6: Diagnosing and managing thyroid dysfunction | □ | □ |
| C7: Diagnosing and managing insomnia / sleep disturbance | □ | □ |
| C8: Treating urinary tract infection | □ | □ |
| C9: Diagnosing and managin chronic obstructive pulmonary disease | □ | □ |
| C10: Diagnosing and managing allergic rhinitis | □ | □ |
| C11: Diagnosing and managing urticaria / angioedema | □ | □ |
| C12: Diagnosing and determining the urgency of headache | □ | □ |
| C13: Appropriate management of hematuria | □ | □ |
| C14: Diagnosing and determining the urgency of dizziness | □ | □ |
| C15: Outpatient management of heart failure | □ | □ |
| C16: Diet therapy in the outpatient encounter | □ | □ |

This is the end of the survey.

Please take a while to review if all items have been marked appropriately.

Thank you very much for your participation.

Appendix

[**SPI scoring system]**

One point is given to the option "Do," whereas "Don't do" is allotted zero points. The scores for total SPI and each subdomain are calculated by the simple sum of points from the questionnaire. A higher SPI score indicates broader scope of practice.
