# Supplementary material for: Development of a questionnaire to measure primary care physicians’ scope of practice
Source: BMC Fam Pract. 2015 Nov 2;16:161. doi: 10.1186/s12875-015-0357-z (PMC4630913; doi:10.1186/s12875-015-0357-z)
Supplement: Additional file 2: — The Scope of Practice Inventory (Japanese version). (DOC 110 kb) [file 12875_2015_357_MOESM2_ESM.doc]

The Scope of Practice Inventory (SPI Japanese version)

本アンケートは、あなたの現在の主な勤務施設における診療内容の幅広さをお尋ねするものです。所要時間は5〜10分程度です。以下の説明をよくお読みいただき、全ての項目へ率直にお答えください。

年齢: 性別: 日付:

◼︎以下の25項目のうち、あなたが「**現在勤務している医療施設で実際におこなっている**内容」はどれでしょうか。おこなっている場合は「実施している」に、おこなっていない場合は「実施していない」を選択して下さい。項目の内容がはっきりわからない場合も「実施していない」を選択してください。

- **実施可能である項目、以前の施設で行なっていた項目でも、現勤務地で実施していない場合は「実施していない」を選択して下さい。**

| 項目 | 実施している | 実施していない |
| --- | --- | --- |
| A1：経鼻胃管の挿入 | □ | □ |
| A2：輸血療法の実施 | □ | □ |
| A3：誤嚥を繰り返す患者に対する経管栄養の適応判断 | □ | □ |
| A4：胸水穿刺の実施 | □ | □ |
| A5：腹水穿刺の実施・結果解釈 | □ | □ |
| A6：動脈血液ガスの採取・解釈 | □ | □ |
| A7：気管挿管 | □ | □ |
| A8：経腸栄養の管理 | □ | □ |
| A9：胃瘻交換、胃瘻関連トラブルへの対応 | □ | □ |
| A10：呼吸不全に対するバッグマスク換気 | □ | □ |
| A11：終末期患者に対するオピオイドを含む疼痛コントロールの実施 | □ | □ |
| A12：終末期の疼痛以外の諸症状(嘔気・せん妄など)への対応 | □ | □ |
| A13：終末期患者に対する疼痛スケールを用いた鎮痛剤調整 | □ | □ |
| A14：頭部CT画像の基本的所見の読影 | □ | □ |
| A15：非癌患者のターミナルケア | □ | □ |
| A16：尿道カテーテルの挿入・留置 | □ | □ |
| A17：ショックバイタルへの初期治療 | □ | □ |
| A18：終末期患者の家族に対する予測される経過の説明 | □ | □ |
| A19：経静脈的鎮静・鎮痛の実施 | □ | □ |
| A20：意識障害への初期対応 | □ | □ |
| A21：延命治療に関するカウンセリング | □ | □ |
| A22：せん妄の診断・治療 | □ | □ |
| A23：腰椎穿刺の適応判断・実施 | □ | □ |
| A24：頭部MRIの基本的所見の読影 | □ | □ |
| A25：脳卒中の初期評価・初期対応 | □ | □ |

◼︎以下の27項目のうち、あなたが「**現在勤務している医療施設で実際におこなっている**内容」はどれでしょうか。おこなっている場合は「実施している」に、おこなっていない場合は「実施していない」を選択して下さい。項目の内容がはっきりわからない場合も「実施していない」を選択してください。

- **実施可能である項目、以前の施設で行なっていた項目でも、現勤務地で実施していない場合は「実施していない」を選択して下さい。**

| 項目 | 実施している | 実施していない |
| --- | --- | --- |
| B1：捻挫の初期治療(シーネ固定を含む) | □ | □ |
| B2：肘内症の整復 | □ | □ |
| B3：熱傷の診断・治療 | □ | □ |
| B4：整形外科疾患に対する日常生活指導 | □ | □ |
| B5：変形性膝関節症の診断・治療 | □ | □ |
| B6：保護者に対する小児の発熱時対応の指導 | □ | □ |
| B7：動物・人咬傷の初療とフォローアップ | □ | □ |
| B8：膝関節穿刺 | □ | □ |
| B9：単純骨折の初期治療(シーネ固定を含む) | □ | □ |
| B10：急性単関節炎の診断・治療 | □ | □ |
| B11：トリガーポイント注射の実施 | □ | □ |
| B12：耳鏡を用いた外耳道・鼓膜の評価 | □ | □ |
| B13：小児の末梢静脈路確保 | □ | □ |
| B14：小児への輸液内容(種類・流速)の指示 | □ | □ |
| B15：胸部外傷に対するバストバンドの適応判断 | □ | □ |
| B16：小児の気道症状に対するレントゲン適応の判断 | □ | □ |
| B17：表在性損傷の止血処置 | □ | □ |
| B18：肩関節周囲炎の診断・治療 | □ | □ |
| B19：前眼部の器具・薬品を用いない診察・評価 | □ | □ |
| B20：鼻出血の止血 | □ | □ |
| B21：急性中耳炎の診断・内服治療 | □ | □ |
| B22：指神経ブロックの実施 | □ | □ |
| B23：裂創の縫合 | □ | □ |
| B24：小児の熱性痙攣の初療、管理方針の決定 | □ | □ |
| B25：耳垢・耳内異物除去 | □ | □ |
| B26：鑑別を考慮した皮疹の診察 | □ | □ |
| B27：スキンケアに関する指導 | □ | □ |

◼︎以下の16項目のうち、あなたが「**現在勤務している医療施設で実際におこなっている**内容」はどれでしょうか。おこなっている場合は「実施している」に、おこなっていない場合は「実施していない」を選択して下さい。項目の内容がはっきりわからない場合も「実施していない」を選択してください。

- **実施可能である項目、以前の施設で行なっていた項目でも、現勤務地で実施していない場合は「実施していない」を選択して下さい。**

| 項目 | 実施している | 実施していない |
| --- | --- | --- |
| C1：気管支喘息の診断・治療 | □ | □ |
| C2：糖尿病の診断・治療 | □ | □ |
| C3：脂質代謝異常症の診断・治療 | □ | □ |
| C4：高血圧の診断・治療 | □ | □ |
| C5：高尿酸血症の診断・治療 | □ | □ |
| C6：甲状腺機能異常症の診断・治療 | □ | □ |
| C7：睡眠障害のプライマリケアとしての評価・治療 | □ | □ |
| C8：尿路感染症の診断と外来治療 | □ | □ |
| C9：慢性閉塞性肺疾患の診断・治療 | □ | □ |
| C10：アレルギー性鼻炎の診断・治療 | □ | □ |
| C11：蕁麻疹／血管浮腫の診断・治療 | □ | □ |
| C12：頭痛の診断と緊急性の判断 | □ | □ |
| C13：血尿患者への適切な対応 | □ | □ |
| C14：めまいの診断と緊急性の判断 | □ | □ |
| C15：うっ血性心不全の外来治療 | □ | □ |
| C16：外来診療中の食事内容へのアドバイス | □ | □ |

以上でアンケートは終了です。

お手数をおかけしますが、最後に**記入漏れ項目**がないか、いま一度ご確認をお願い致します。

ご協力いただき、まことにありがとうございました。

補足資料

　【**SPI スコアリングシステム】**

“実施している”には１点、"実施していない"には０点が配分されます。

SPI合計スコアならびに各下位尺度スコアは、各構成項目のポイント合計で算出されます。

スコア得点が高いほど、より診療範囲が広いことを示します。
